# Insulin Production and Resistance in Different Models of Diet-Induced Obesity and Metabolic Syndrome

**DOI:** 10.3390/ijms18020285

**Published:** 2017-01-28

**Authors:** Salamah M. Alwahsh, Benjamin J. Dwyer, Shareen Forbes, David H. van Thiel, Philip J. Starkey Lewis, Giuliano Ramadori

**Affiliations:** 1 Clinic for Gastroenterology and Endocrinology, University Medical Center, Georg-August-University Goettingen, Goettingen D-37075, Germany; 2 MRC Centre for Regenerative Medicine, University of Edinburgh, Edinburgh EH16 4UU, UK; bdwyer@exseed.ed.ac.uk (B.J.D.); pstarkey@exseed.ed.ac.uk (P.J.S.L.); 3 Endocrinology Unit, University/BHF Centre for Cardiovascular Science, Queen’s Medical Research Institute, University of Edinburgh, Edinburgh EH16 4TJ, UK; Shareen.Forbes@ed.ac.uk; 4 Advanced Liver and Gastrointestinal Disease Center, Chicago, IL 60611, USA; dvanthiel@dr.com

**Keywords:** alcohol, insulin resistance, type II diabetes mellitus (T2DM), obesity, fatty liver

## Abstract

The role of the liver and the endocrine pancreas in development of hyperinsulinemia in different types of obesity remains unclear. Sedentary rats (160 g) were fed a low-fat-diet (LFD, chow 13% kcal fat), high-fat-diet (HFD, 35% fat), or HFD+ 30% ethanol+ 30% fructose (HF-EFr, 22% fat). Overnight-fasted rats were culled after one, four or eight weeks. Pancreatic and hepatic mRNAs were isolated for subsequent RT-PCR analysis. After eight weeks, body weights increased three-fold in the LFD group, 2.8-fold in the HFD group, and 2.4-fold in the HF-EFr (*p* < 0.01). HF-EFr-fed rats had the greatest liver weights and consumed less food during Weeks 4–8 (*p* < 0.05). Hepatic-triglyceride content increased progressively in all groups. At Week 8, HOMA-IR values, fasting serum glucose, C-peptide, and triglycerides levels were significantly increased in LFD-fed rats compared to that at earlier time points. The greatest plasma levels of glucose, triglycerides and leptin were observed in the HF-EFr at Week 8. Gene expression of pancreatic-insulin was significantly greater in the HFD and HF-EFr groups versus the LFD. Nevertheless, insulin: C-peptide ratios and HOMA-IR values were substantially higher in HF-EFr. Hepatic gene-expression of insulin-receptor-substrate*-1/2* was downregulated in the HF-EFr. The expression of phospho-ERK-1/2 and inflammatory-mediators were greatest in the HF-EFr-fed rats. Chronic intake of both LFD and HFD induced obesity, MetS, and intrahepatic-fat accumulation. The hyperinsulinemia is the strongest in rats with the lowest body weights, but having the highest liver weights. This accompanies the strongest increase of pancreatic insulin production and the maximal decrease of hepatic insulin signaling, which is possibly secondary to hepatic fat deposition, inflammation and other factors.

## 1. Introduction

The prevalence of non-alcoholic fatty liver disease (NAFLD) is 80% in obese adults, 30%–50% in patients with type 2 diabetes mellitus (T2DM), up to 90% in patients with hyperlipidemia [1,2], and 19% in non-obese subjects [3]. A high prevalence of liver steatosis is not only in subjects with T2DM, but also preceding the diagnosis of diabetes [4,5]. The definition of NAFLD allows for a daily alcohol consumption of <30 g [6], and dietary fructose is increasingly consumed. Factors responsible for hyperglycemia and hyperinsulinemia are not fully understood.

Both insulin clearance and insulin sensitivity correlate inversely with dietary fat/carbohydrate ratio [7,8]. Data suggest that increased liver fat impairs insulin clearance and hepatic and adipose tissue insulin resistance (IR) characterizes of T2DM [9]. Serum insulin concentrations are significantly higher in subjects with T2DM [9]. It has been proposed that this hyperinsulinemia is the result of both increased insulin production and decreased insulin clearance [7,10]. Hepatic uptake of insulin is incompletely understood but appears to be a receptor-mediated process [11]. Free fatty acids (FFAs) inhibit insulin uptake, degradation and action in the liver [8]. NAFLD could inhibit hepatic insulin clearance in patients, which is also associated with reduced insulin sensitivity [12]. Indeed, partial ablation of hepatic ApoA5 in mice fed a high-fat diet (HFD) reduced triglyceride accumulation in liver, skeletal muscles, and protected them from HFD-induced IR [13].

Many glucose-lowering anti-diabetic drugs enact their pharmacologic effects on the liver [11]. The insulin-sensitizer agonist, PPAR-γ, stimulates adiponectin production, which in turn decreases hepatic steatosis by activating AMP-activated protein kinase, enhancing insulin sensitivity [14]. Glyburide administration has been demonstrated to increase hepatic insulin extraction [15], as do rosiglitazone and metformin [16]. A reduced expression of the insulin receptor and insulin receptor substrate-1/2 (IRS-1/2) is associated with impairment of insulin-signaling and expression of *Irs-1/2* in NASH subjects is markedly lower than that seen in healthy individuals [17].

An association between sugar availability and the prevalence of T2DM has been reported, independent of its role in obesity [18]. Chronic consumption of a “Western” diet, characterized by sugar and saturated fat-rich foods, triggers the development of T2DM [19]. This has been verified in human individuals subjected to a high-fructose diet for 7 days led to an increased lipid deposition in liver and muscle, and reduced hepatic insulin sensitivity [20,21]. Similarly, acute alcohol ingestion promotes the hepatic expression of lipogenic and pro-inflammatory genes and lipid peroxidation [22]. However, it is unclear whether hepatic steatosis directly causes T2DM, or metabolic dysfunction is responsible for steatosis, or an interaction of both is regulated to produce T2DM. On the other hand, Mediterranean diet has shown notable benefits to ameliorate the risk factors associated with metabolic syndrome and NAFLD in humans [23]. The main features of the Mediterranean diet acting on metabolism are represented by its whole-grain and low glycemic index cereal-based items, its FFAs profile (unsaturated), and its content in phytochemical compounds [23].

A few studies have examined the effects of concurrent intake of alcohol and fructose on liver injury, obesity and MetS. Although simultaneous exposure to ethanol and fructose has been studied acutely in volunteers [24,25], in mice [26], in primary-mouse hepatocytes [27], and we have examined the effects of longer-term consumption (four-week pilot study) of alcohol and fructose in rats [28], the dynamics of metabolic change have not fully been assessed at short- and long-term courses. Additionally, changes in pancreatic hormone production have not been evaluated in these conditions. To address these shortcomings, we investigated longitudinally the short- and long-term effects of concurrent alcohol and fructose consumption on the onset of obesity, NAFLD, MetS and diabetes among young rats. In parallel, we studied insulin gene-expression in pancreatic tissue and the expression of *Irs-1/2* and other markers in the hepatic tissue.

## 2. Results

### 2.1. Chronic Consumption of Ethanol-Plus-Fructose-Enriched-HFD (HF-EFr) Induced Less Weight Gain Than LFD, But Increased Liver Weight and Visceral Fat

**Figure 1:**
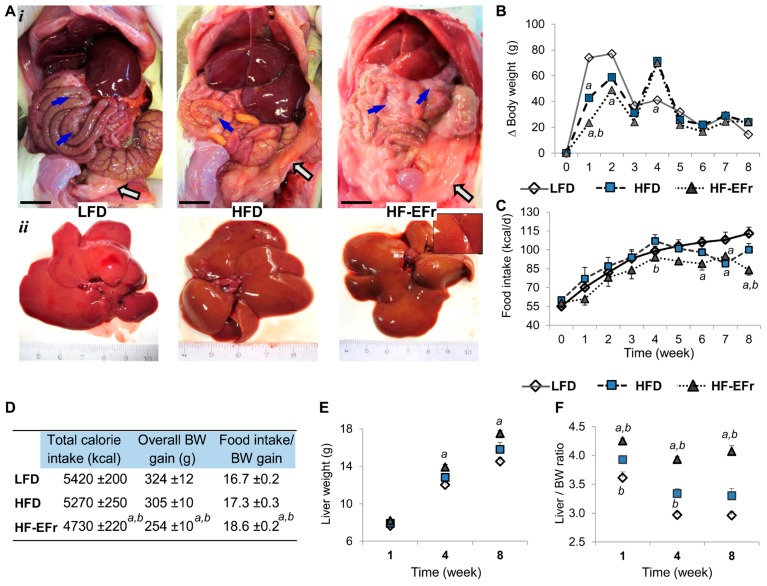
Changes in rats’ bodyweight (BW), liver weight and liver-to-BW ratio. (**A*i***) Representative images of gross appearance of liver and intraabdominal adipose tissue. Ethanol+ fructose-diet (HF-EFr) induces an increased mesenteric fat depots that wrapping of the intestine (blue arrows) than those fed the high-fat-diet (HFD) alone or low-fat-diet (LFD, chow) at Week 8. (**A*ii***) Gross morphology of dissected livers. Inset represents higher magnification; (**B**) Stacked columns show weekly BW gain of each animal group; (**C**) Changes in the average daily energy intake of each group per week; (**D**) Overall food intake and BW gain throughout the eight weeks experiment; (**F**) Liver-weight and (**E**) liver-to-BW ratio. Results represent mean ± SEM (*n* = 6). Rats were fed the LFD that contains 13% fat + 58% carbohydrate (CARB (together 71%)), HFD 35% fat + 48% CARB (83%), or HF-EFr, 22% fat + 37% CARB (59%). Superscript letters represent significant difference amongst groups at assigned time point, while the symbols represent the difference amongst time points within the same group: *^a^* *p* < 0.05 vs. the LFD, *^b^* *p* < 0.05 vs. HFD group. Bar = 1 cm. Open square depicts changes occurred to LFD-fed rats, filled square represents the HFD group, whereas triangular symbol represents the HF-EFr group. *n* = 4–6 rats/group per time point.

We performed an initial macroscopic assessment of fat deposition, liver appearance and general condition of animals. Epididymal-fat pads of rats fed a HFD or HF-EFr diets appeared larger compared to the LFD, and the livers of these rats appeared pale (Figure 1A). Body weight (BW) gain was also restricted in rats fed a HF-EFr-diet compared to rats fed LFD or a HFD (Figure 1B). In the first four weeks of feeding, all rats had gained 67% of their final BWs and consumed 46% of total energy recorded at Week 8. Overall, energy consumption was comparable amongst experimental groups at Week 1. However, overall calorie intake was reduced in HF-EFr-fed rats (2156 ± 147 kcal/rat) compared to LFD (2410 ± 150 kcal/rat; *p* < 0.05) and HFD (2560 ± 190 kcal/rat; *p* < 0.05) at Week 4. Over the 8 weeks, the HF-EFr-fed rats consumed less energy than other groups (Figure 1C), and consumed 10.0 ± 0.3 g·kg^−1^·day^−1^ ethanol per rat.

We also looked at how each diet affects the animal’s BW gain. Thus, amount of calories of each diet that is required to increase the animal’s BW by 1 g has been calculated. Rats fed the HF-EFr diet needed ~10% more energy than that needed by the LFD-fed or HFD-fed rats to raise their BW by 1 g (*p* < 0.05, Figure 1D). The HF-EFr diet induced a significant increase in animals’ liver weights compared to the LFD at Weeks 4 and 8 (Figure 1E). Liver-to-BW ratio was the greatest in the HF-EFr group (Figure 1F, *p* < 0.01). Together, chronic ad libitum energy intake (in the LFD or HFD group) significantly increased BW independent of specific diet composition. Long-term overfeeding of HF-EFr diet increased liver weight and visceral adipose tissue.

### 2.2. Effects of Short- and Long-Term Diet Consumption on the Levels of Glucose Homeostasis

**Figure 2:**
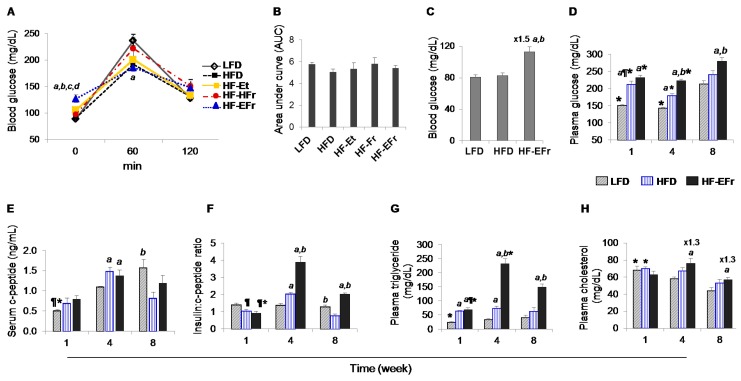
Changes in blood metabolic parameters. (**A**) Blood glucose levels obtained after 16 h fasting during *intraperitoneal* glucose tolerance test of 13 week-old rats after 3 weeks of feeding; and (**B**) corresponding total cumulative glucose levels for each treatment group are summed as “area under the curve” (AUC); (**C**) Baseline measurement of 16 h fasted 9 week-old rats reared for two weeks on respective diets; (**D**) Plasma glucose levels at time of cull after 10–12 h fast; (**E**) Insulin:C-peptide ratio; and (**F**) C-peptide; (**G**) triglycerides; and (**H**) cholesterol levels at time of sacrifice. *^a,b,c,d^* indicate significant difference between HF-EFr and other groups: *^a^* LFD: low-fat-diet, *^b^* HFD: high-fat-diet, *^c^* HF–Et: HFD + 30% kcal ethanol, *^d^* HF–HFr: HFD + 30% kcal fructose, while ^¶^ significant difference vs. corresponding Week 4 of each group and * represents significant difference vs. matched Week 8 of the same group (difference among time points). Tukey’s and unpaired *t*-tests were used for statistical analysis.

In two different separated experiments, basal glucose levels were above 100 mg/dL (diabetic) in the HF-EFr fed rats (Figure 2A,C). However, after 60 min of glucose administration, blood glucose levels were increased greatly in this group. No significant difference in glucose tolerance was observed among overall change of blood glucose, as calculated by the rate of change in blood glucose levels (AUC, Figure 2B). Younger HF-EFr-fed rats had also shown the highest fasting glucose levels after two weeks of feeding (*p* < 0.05, Figure 2C).

Fasting plasma glucose levels were increased by 140% higher at Week 8 compared to Week 1 or 4 in the LFD-fed rats (*p* < 0.05, Figure 2D). Analogous findings were seen in the other groups. At all time points, concurrent consumption of alcohol and fructose-containing diet significantly increased the fasting levels of plasma glucose compared to the LFD or HFD groups.

HOMA-IR values in the LFD-fed rats were significantly higher at Week 8 as compared to values observed at earlier time points (Table 1). HF-EFr-fed rats displayed increased insulin resistance indices compared to the LFD or HFD. In addition, animals in the HF-EFr group also exhibited reduced insulin sensitivity as demonstrated by QUICKI analysis (Table 2). C-peptide levels were elevated after four weeks of HFD or HFD-EFr diets (Figure 2E). However, after eight weeks, C-peptide levels were significantly greater in the LFD group compared to the HFD. The highest insulin-to-C-peptide ratios were observed in the HF-EFr-fed rats at Weeks 4 and 8 (*p* < 0.05 vs. other groups). In the LFD, fasting glucose-to-insulin ratios were significantly lower at Week 8 compared to that at Week 1 (Figure S1).

### 2.3. Changes in Metabolic Parameters and Liver Enzymes

Markers for liver injuries including plasma albumin production and plasma transaminases, kidney function test and other metabolic biomarkers were assessed. Levels of plasma albumin were the lowest in the HF-EFr-fed rats at Weeks 1 and 8 (*p* < 0.05), reflecting decreased hepatic synthesis in this group (Table 2). Serum transaminases activities were the greatest in the HF-EFr group at Week 4 and 8 (Table 2). In all groups, plasma creatinine levels increased over time but remained normal, while the activity of pancreatic amylase decreased (Table 2). Fasting serum leptin levels reflected the progressive increase in BW and the increased visceral and epididymal fat observed in the groups.

Fasting plasma triglyceride levels increased significantly in the HF-EFr-fed animals during the 8 weeks versus the other groups (Figure 2G). HF-EFr-fed rats exhibited greater cholesterol levels at later time points compared to age-matched LFD- or HFD-fed rats (*p* < 0.05, Figure 2H).

### 2.4. Histology of the Liver and the Onset of Hepatic Steatosis

**Figure 3:**
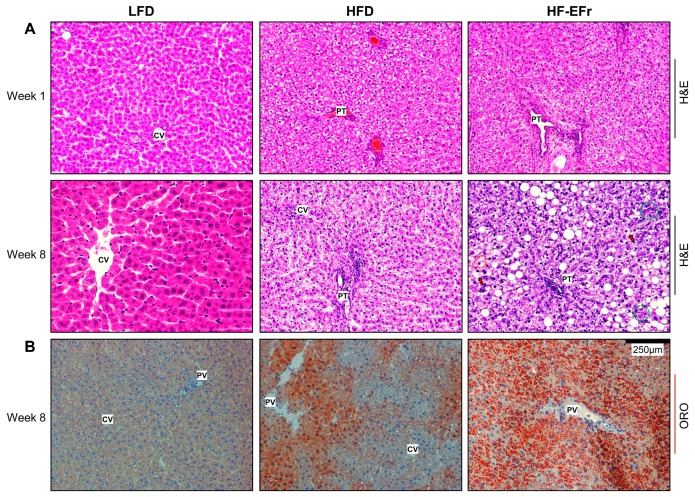
Characterization of hepatic lipid accumulation. (**A**) Representative images of H&E staining show areas of macrosteatosis (white vesicles) surrounding the portal triad (PT) in the livers of the HF-EFr-fed rats. The HF-EFr diet induced microsteatosis. Mallory’s hyaline bodies (purple filamentous structure) are present in ballooned hepatocytes with red-dashed circumference in the HF-EFr livers. Multiple small, well defined vacuoles instead of the large single vacuoles present within the hepatocytes give the cytoplasm a foamy appearance (arrows). Green circles indicate microgranulomas (inflammatory clusters). The large crisp vacuoles (a coalescence of small vacuoles) which often displace the nucleus to periphery and give the hepatocytes the appearance of adipocytes. The small vacuoles may coalesce to form the large ones; (**B**) Panel of Oil Red O (ORO) staining at Week 8 of the experiment (×100) demonstrates increased fat accumulation (orange-red vesicles within hepatocytes) mainly in the HFD and HF-EFr groups. CV: central vein.

The LFD-fed rats exhibited normal liver architecture despite increased intrahepatic triglyceride content at Weeks 4 (150%) and 8 (170%) compared to observations at Week 1 (Figure 3A). One week of animals’ feeding, both HFD and HF-EFr-diet induced fat droplet deposition. At this time point, fasting serum C-peptide levels, insulin-to-C-peptide ratio or HOMA-IR values were comparable among the groups. Heterogeneous phenotypes of lipid droplets were observed: micro- and macro-steatosis predominantly in the portal triad of HFD-fed rats and discrete lipid droplets evenly distributed in the livers of HF-EFr-fed rats (Figure 3B). Chronic feeding of the HF-EFr induced severe steatosis associated with mild foci of inflammation. Moreover, cytoplasmic Mallory’s bodies appeared in the liver sections of the HF-EFr-fed rats. The hepatic triglyceride content of the HFD and HF-EFr groups were comparable and substantially higher than observed in the LFD group. Still, long-term feeding with the LFD *ad libitum* elicited progressive hepatic fat deposit (Figure S2A–D).

### 2.5. Alterations of mRNA Expression in the Liver and Pancreas

**Figure 4:**
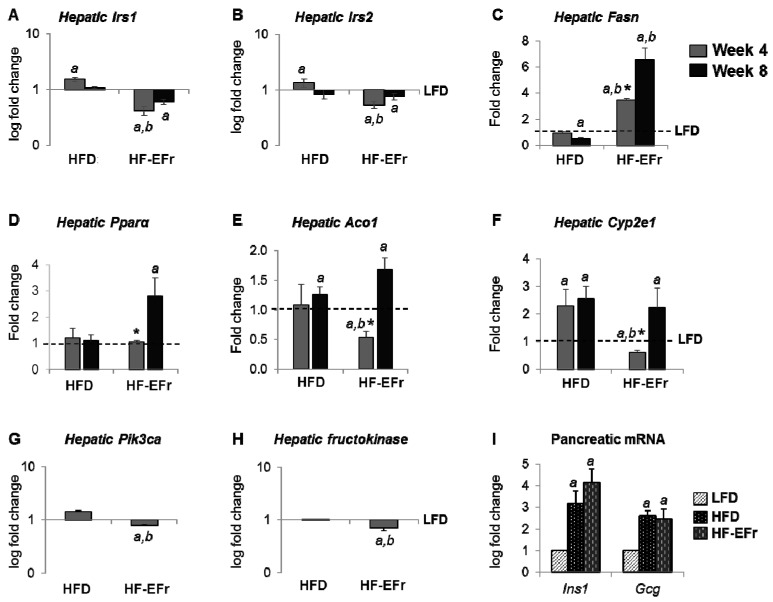
Changes in mRNA expression in rat liver and pancreas. (**A**–**H**) Relative gene expression of insulin receptor substrate (*Irs*)-1 and -2, fatty acid synthase (*Fasn*), peroxisome proliferator-activated receptor-α (*Ppara*), acyl CoA oxidase 1 (*Aco1*), and cytochrome P450 2E1 (*Cyp2e1*) in liver tissue of each group at Weeks 4 and 8. The expression of phosphatidylinositol-4,5-bisphosphate 3-kinase, catalytic subunit α (*Pik3ca*) and *fructokinase* was measured at Week 4. (**I**) Insulin-1 (*Ins1*) and glucagon (*Gcg*) mRNA expression in pancreas at Week 4. LFD gene expression was set at 1 on the y-axis by a dashed line. *^a^* *p* < 0.05 vs. the LFD group, *^b^* *p* < 0.05 vs. the HFD, * vs. corresponding Week 8 of each group. Data are fold differences in relation to value of the LFD (control) (dashed line).

Hepatic mRNA expressions of *Irs1/2* were significantly reduced in the HF-EFr group compared to LFD or HFD groups (Figure 4A,B). The HF-EFr diet significantly increased the expression fatty acid synthase (*Fasn*) by 3.8-fold at Week 4 and 6.2-fold at Week 8 as compared to the LFD or HFD (Figure 4C). The HFD significantly diminished lipogenic genes at Week 8 (*p* < 0.05 vs. the LFD). A significant increase in the expression of key genes regulating fat β-oxidation is observed predominantly in the HF-EFr group at Week 8 (Figure 4D–F).

Phosphatidylinositol-4,5-bisphosphate 3-kinase, catalytic subunit α (Pik3ca) is involved in the activation of Akt1 upon stimulation by receptor tyrosine kinases ligands such as insulin and IGF1, and in downstream signaling via IRS proteins. The *Pik3ca* and *fructokinase* mRNA transcripts were significantly reduced in the livers of HF-EFr-fed rats (Figure 4G). Pancreatic RNA was extracted from rats at four weeks. Both HFD and HF-EFr diets upregulated *insulin-1* (*Ins1*) and glucagon (*Gcg*) transcripts (Figure 4I). These data demonstrate that concomitant intake of ethanol and fructose impairs in insulin-signaling pathways and increases the risk of oxidative stress.

### 2.6. Consumption of Alcohol-Plus-Fructose-Containing HFD Progressively Induces an Activation of Extracellular Signal-Regulated Protein Kinases-1 and -2 (ERK1/2)

**Figure 5:**
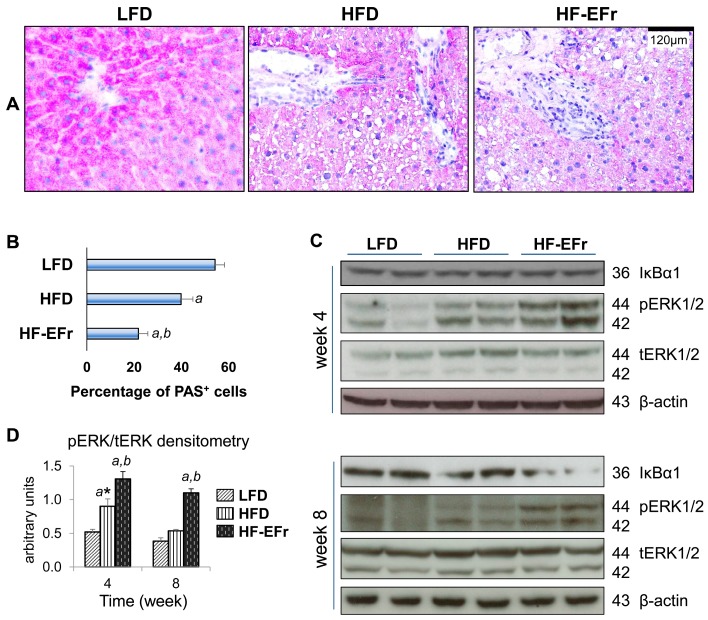
Histological features of the liver based on PAS staining. (**A**) Representative micrographs of PAS at Week 8 display densely glycogen deposits (pink/purple areas) in cytoplasm of the hepatocytes; (**B**) The number of PAS^+^ cells is expressed as percentage of the total number of counted cells in high-power-field; (**C**) Representative immunoblots of IκBα1, phosphorylated and total ERK1/2; (**D**) Densitometric analysis for bands intensity of pERK1/2 to tERK1/2. *^a^* *p* < 0.05 vs. the LFD group, *^b^* *p* < 0.05 vs. the HFD, * vs. Week 8 of each group.

Hepatic sections of LFD-fed rats displayed centrilobular deposition of glycogen (Figure 5A). Number of periodic acid schiff (PAS^+^) hepatocytes decreased significantly in the HFD group. Despite development of hyperinsulinemia, the lowest number of PAS^+^ hepatocytes was observed in HF-EFr group (Figure 5A,B).

The cellular effect of alcohol and fructose uptake on activation of the NF-κB pathway was explored by determining the effect on IκBα degradation (an inhibitor for NF-κB, and a prerequisite step to initiate inflammatory gene transcription). Both HFD alone and HF-EFr diet increased the phosphorylation of ERK1/2 at Week 4. At this time point, no significant change in the IκBα protein expression was observed among the groups (Figure 5C,D). In contrast, eight weeks feeding with HF-EFr significantly provoked the phosphorylation of ERK1/2 and reduced IκBα expression as compared to the HFD alone or LFD (Figure 5C).

## 3. Discussion

The findings of these studies suggest that both the quantity of daily consumed energy and quality of diet together with sedentary life-style are important determinants for the induction of an overweight body composition, intrahepatic fat accumulation and hyperglycemia. Although HF-EFr-fd rats consumed less energy and had lower BWs, they had the highest liver weights, and exhibited hyperinsulinemia and worst markers of MetS, suggesting that the HF-EFr is a pathologic diet. Furthermore, the HF-EFrdiet increased the expression of inflammatory regulators, attenuated expression of insulin-signaling regulatory genes, and disrupted insulin clearance.

### 3.1. Obesity, Calorie Consumption and Liver Weight

Rats gained 2/3 of their BWs in the first 4 weeks of the study. Adding ethanol+ fructose to the HFD makes the diet energy-rich but nutritionally poor (e.g., containing less protein), similar to the “Western” diet. The frequent consumption of an empty-caloric diet, without physical activity, increases fat storage and results in obesity, hepatomegaly and T2DM [29]. Indeed, in the HF-EFr group, visceral fat was more pronounced and serum leptin levels were higher, similar to changes reported in patients [30]. Liver weight was highest in response to the HF-EFr-diet, and was associated with a significant progressive increase in intrahepatic triglyceride content as a result of expression of *Fasn*. These results coincide with previous findings that showed long-term fructose overfeeding increases fat accumulation in adipose tissues and liver, findings associated with an increased risk of diabetes [31,32]. In addition, ethanol has been found to induce perivenular fibrosis and an enlargement of hepatocyte size, resulting in the enlarged liver [33,34]. These results suggest the existence of multiple forms of obesity with different underlying pathological mechanisms.

### 3.2. Consumption of Ethanol-Plus-Fructose and Liver Injury

The Lieber-DeCarli liquid diet is an efficient procedure to study the effects of ethanol under controlled nutritional conditions. This diet allows for alcohol consumption of clinical relevance and offers flexibility to adjust to special experimental or physiologic needs by allowing for various substitutions required for a particular experimental design, including changes in lipids, carbohydrates or other dietary constituents [35]. In contrast, alcohol administration in water is associated with insufficient consumption and heavy weight loss [22,36]. In our current study, we have found that rats of HF-EFr group consumed 10.0 g ethanol/kg BW/day per rat. This amount is sufficient to drive hepatic steatosis, as average ethanol intake in liquid HFD containing 35% kcal alcohol ranges from 10 to 14 g/kg/day in rats [35]. The difference in duration and frequency of alcohol consumption between humans and animals should be considered [37]. For example, a human could drink 3–5 drinks in 2 h, whereas in these experiments rats consumed the alcoholic diet within 24 h.

Unlike HFD group, the HF-EFr group developed severe steatosis associated with mild inflammation consisting of focal microgranuloma and Mallory’s bodies found in ballooned hepatocytes. These findings correlated with increased serum ALT activity, decreased plasma albumin levels, and increased levels of various inflammatory regulators including: NF-κB, lipocalin-2, PAI-1, CD68, and serum CD14. The markers for liver injury were greater in a prior study [28], i.e., rats were fed on chow of 10 weeks before they were fed the HF-EFr diet for additional four weeks. This is probably due to prolonged *ad libitum* calorie intake increased liver intolerance and sensitivity for ethanol and/or fructose effects. A significant decrease in hepatic IκBα1 protein in the HF-EFr-fed rats was observed. The activation of NF-κB and TLR4-CD14 cascade presumably resulted from an activation of CD68^+^ macrophages, as the chronic intake of HF-EFr enhances the influx of endotoxin to portal blood [26,38,39].

Furthermore, the ingestion of the HF-EFr diet led to a substantial upregulation of the expression of key genes involved in peroxisomal (*Aco1 and Pparα*) and microsomal (*Cyp2e1*) FFA β-oxidation, which are known to increase production of reactive oxygen species (ROS). The expression of *Fans*, which catalyses FFA synthesis was upregulated in the HF-EFr group. Activation of β-oxidation and lipogenesis simultaneously in the livers of HF-EFr group is analogous to the features observed in NASH patients [40,41].

The acute interaction between ethanol and fructose has been studied in healthy volunteers [24]. The administration of fructose after ethanol resulted in an increased formation of phosphomonoesters and a depletion of ATP [24,42], subsequently increasing uric acid production, which induces mitochondrial oxidative stress [43]. Indeed, the highest levels of uric acid were found in the HF-EFr group. Increased uric acid levels in liver and serum is a consequence of fructose metabolism, and an indirect evidence for oxidative stress [43].

**Figure 6:**
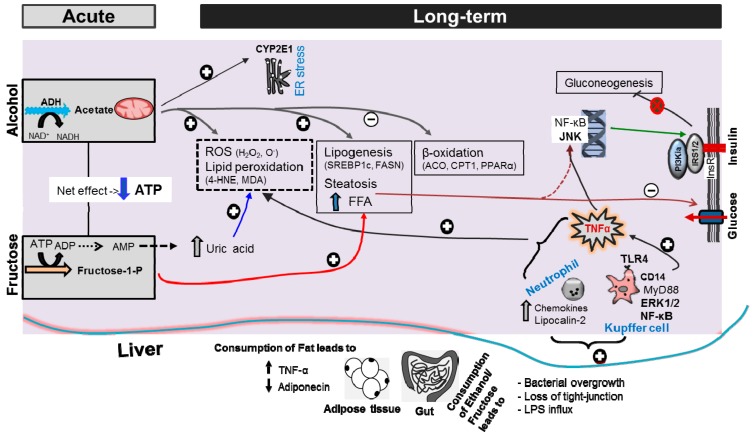
Hypothetical illustration of potential underlying mechanism by which ethanol and fructose could cause pathological effects. Alcohol and fructose are metabolized in hepatocyte. Acute consumption of alcohol leads to production of NADH and acetate. Acute fructose consumption causes ATP depletion during fructose phosphorylation. Prolong ATP depletion results in an increased uric acid production which exacerbates ROS formation. Chronic ethanol intake leads to accumulation of acetate, which activates SREBP1c (lipogenesis) and decrease of expression of PPARα (β-oxidation of FFA). Acetate oxidation in the mitochondria results in the formation of ROS and glutathione depletion. Acetate oxidation via CYP2E1 causes endoplasmic reticulum (ER) stress. Chronic fructose intake causes mitochondrial dysfunction and hepatic steatosis. By binding TLR4, LPS activates Kupffer cells via signaling cascade involving CD14, and the activation of ERK1/2, JNK. LPS amplifies neutrophils recruitment in the liver. Elevated production of TNFα by adipose tissue decreases sensitivity to insulin. Free fatty acids (FFA) are also implicated in the etiology of obesity-induced insulin resistance. Both TNFα and FFA are potent JNK activators. Consequently, uptake of insulin and glucose is reduced and resulting less inhibition of gluconeogenesis. Carnitine palmitoyltransferase 1 (CPT1), ACO: acyl-CoA oxidase, 4-HNE: 4-hydroxynonenal, MDA malondialdehyde, TLR4: toll-like receptor-4. Upward or downward arrows indicate “stimulate” or “ameliorate”, respectively, similar to “+” or “–“. The red cross indicates “inhibits”.

The exposure to ethanol and fructose has been reported to increase ROS production in the liver, accompanied by an activation of stress/inflammation markers (e.g., ERK1/2, and an increased degradation of IκBα). ERK1/2 could phosphorylate downstream inflammatory transcription factors, e.g., JNK and Smad1-4 [44]. The HF-EFr diet significantly increased the phosphorylation of hepatic ERK1/2, which was associated with hyperglycemia and an increased insulin-to-C-peptide ratio. ERK1/2 activity is influenced by various hormone (insulin and leptin) and diet compositions [45,46]. Fructose caused an impairment of glucose utilization and insulin-signaling through ROS-mediated activation of JNK and ERK1/2 pathways, which can be denied by administration of antioxidants [47]. Furthermore, TNFα and JNK play central roles in obesity and IR [48]. The present data on liver injury are in accordance with the studies which demonstrate that mitochondrial dysfunction progressively contributes to the development of NAFLD (Figure 6).

### 3.3. MetS, IR and Insulin Clearance

The HFD alone induced obesity-associated with hepatic steatosis, hyperleptinemia, and only transient increases in HOMA-IR values, C-peptide levels, hypertriglyceridemia, and reduced glucose-to-insulin ratio. These parameters were increased in the LFD-fed rats at Week 8, thus minimizing the effects provided by the other regimens at this time point, similar to prior studies [19]. In fact, the endogenous-fructose production in the LFD and HFD groups could not be excluded. Indeed, a high amount of fructose was observed in the livers of HFD-fed mice, suggesting endogenous-fructose production [43] via aldose reductase/polyol pathway [49]. Mice that were unable to metabolize fructose were protected from increased visceral obesity, fatty liver, hyperinsulinemia and hyperleptinemia after exposure to 10% glucose. Therefore, endogenous-fructose generation in the liver represents an important mechanism by which glucose promotes the development of MetS [49]. Basal glucose levels were greatest in the HF-EFr group; but this was not the case after 60 min of glucose administration. Fasting glucose best reflects hepatic glucose output whereas the 60 min value is more indicative of glucose uptake by the muscle.

The recent data demonstrate that glycogen storage is reduced in steatotic livers, triggered either by the HFD alone or HF-EFr, which is comparable with results of prior reports [19,43]. Conditions that could enhance peripheral glucose levels include the (1) inability of insulin to suppress gluconeogenesis and glucose production [50]; (2) decreased insulin uptake/recognition by (fat-loaded) hepatocytes [11]; and (3) reduced glycogen storage due to increased oxidative stress through a NADPH oxidase pathway [19,51]. Indirect glycogen synthesis through the conversion of 3-carbon intermediates to glucose-6-phoshate could also be responsible [50]. After eight weeks of HF-EFr consumption, reduced glycogen storage in HF-EFr group was associated with increased evidence of oxidative stress, as manifested by increased expression of *Cyp2e1* and *Aco1*. Consumption of HFD or high-sucrose-diet for one week resulted in hepatic IR, and after 5 weeks these diets produced both hepatic and peripheral (skeletal muscles) IR [50]. In the recent study, after one week of feeding, both HFD and HF-EFr diet induced hepatic fat deposition. But fasting serum C-peptide levels, glucose-to-insulin ratio, insulin-to-C-peptide ratio or HOMA–IR values were comparable among the groups at this time point, suggesting that IR is a consequence of the onset of hepatic steatosis. The HF-EFr diet also resulted in a down-regulation of *Irs-1/2* and *Pik3ca* mRNA transcripts, paralleling the findings of Spruss et al. [52].

Insulin-to-C-peptide ratio and glucose levels were increased significantly in the HF-EFr. These data can be explained in part by decreased hepatic insulin extraction as renal function was normal. Indeed, a negative correlation between hepatic insulin extraction and hepatic glucose output has been demonstrated [53]. Reduced insulin clearance was also documented in patients with IR [54]. Prolonged increases in portal insulin levels also result in reduced clearance of insulin due to receptor down-regulation [11]. The present results strongly suggest that hepatic insulin extraction is impaired by chronic consumption of HF-EFr diet, indicating that the liver is an important site for the regulation of insulin extraction, thereby controlling peripheral insulin delivery [53].

Beneficial effects of alcohol drinking [55] including enhancing insulin sensitivity and adiponectin levels in postmenopausal women [56], and preventing the onset of NAFLD in *ob*/*ob* mice have been reported [22,57]. Consumption of HFD decreased adiponectin expression in rodents [58]. Similarly, chronic fructose intake significantly decreased plasma adiponectin levels in rats [59], as HF-Et liquid diet did [60]. The current study indicates that reduced hepatic insulin clearance is not the only mechanism responsible for hyperinsulinemia, but also increased insulin gene-expression parallels the increase of serum glucose levels. Interestingly, Mediterranean diet has been prescribed as a therapeutic option for obese with NAFLD and diabetic patients [61,62].

## 4. Materials and Methods

### 4.1. Animals

Experiments were performed in compliance with the “German Law on the Protection of Animals and the Institutional Guidelines”, and treatment of rats was approved by the local animal ethics committee in the University of Göttingen. All efforts were made to minimize animal suffering, and to reduce the number of used animals, and thus complying with the commonly-accepted “3Rs”.

### 4.2. Induction of Fatty Liver and Animal Sacrifice

Seven-week-old (~160 g) male Sprague-Dawley rats (Charles River, Sulzfeld, Germany) were randomly distributed into five groups (*n* = 4–6 rats/group/time point) and fed either (i) pellet low-fat-diet (LFD, 13% kcal fat, 58% CARB (starch), 29% protein); (ii) Liquid high-fat-diet (HFD) contains 35% fat, 48% CARB (maltose dextrin), 17% protein); (iii) HFD+30% kcal ethanol (3.3% *w*/*w*, HF–EtOH); (iv) HFD+ 30% fructose (HF–HFr); or (v) HFD+ 30% EtOH+ 30% Fr (HF-EFr, 22% fat, 37% CARB (30% Fr–derived energy), 11% protein) for one, four, or eight weeks. More details about diet composition are present in Table 1. Seven days before starting the experiment, concentration of the ETOH solutions was gradually increased in Lieber-DeCarli diet (1%, 5%, 10%, 20% and 25%), allowing the rats to adapt its taste. The HFD rats were pair-fed diets that isocalorically substituted maltose dextrins for ethanol. Animals were housed individually in standard cages and remained sedentary. Using liquid diet for administration of alcohol helps to mimic the effects of alcohol intake in humans. This model allows the investigating of chronic alcohol intake without irrelevant harmful effects [36]. Maltose dextrin of the HFD was isocalorically replaced with ethanol and/or fructose.

Based on our previous findings in a pilot, one time point study [28], the current study focused on the effects of the LFD, HFD, and HF-EFr groups. The latter have shown interesting findings that worth to be studied further at short- and long-term in younger animals. The diets were prepared fresh daily to minimize the evaporation of alcohol and/or change in the diet’s flavor. Animals were fasted overnight before sacrifice using Na-pentobarbital anesthetic overdose (Merial, Hallbergmoos, Germany). Food intake was recorded daily and body weight (BW) was recorded weekly.

### 4.3. Glucose Tolerance Test and Baseline Fasting Glucose

Two different experiments were performed (Figure S3). *Experiment*-*1*: 10 weeks old male Sprague–Dawley rats were fed the five different diets as described above for four weeks. After three weeks, an intraperitoneal glucose tolerance test (iGTT) was performed after a 16 h fast. Rats received 1 g/mL glucose/kg *i*.*p*. Subsequent blood samples were taken from the tail vein at *t* = 0, 60, and 120 min after the glucose injection. Blood glucose was measured using a handheld glucometer (*One Touch-*Ultra). *Experiment*-*2*: rats (7 weeks) were fed LFD, HFD or HF-EFr-diet up to eight weeks. A baseline (*t* = 0 min) fast blood glucose levels were measured after two weeks of feeding.

### 4.4. Biochemical Analyses

At the end of each time point, plasma (heparinized) and serum (in plain tubes) samples were collected from the animals after an overnight fast. The plasma concentration of glucose, triglycerides, total cholesterol, total protein, albumin, creatinine, pancreatic amylase, and plasma transaminases activities were determined utilizing the automated systems in the Clinical Chemistry Institute, Goettingen, Germany.

### 4.5. Radioimmunoassay (RIA)

Serum insulin, C-peptide and leptin levels were measured by the RIA kit, according to the manufacturer’s protocol (Millipore, Billerica, MA, USA). Insulin:C-peptide ratio (insulin and C-peptide are secreted in equimolar quantities by the β-cells; an insulin:C-peptide ratio >1 may therefore indicate reduced insulin clearance), glucose-to-insulin ratios, homeostatic model assessment-insulin resistance (HOMA-IR) index and the quantitative insulin sensitivity check index (QUICKI) were calculated as previously defined [63].

### 4.6. Histopathological Studies

#### 4.6.1. H&E Staining

Five-micrometer thick paraffin-embedded liver samples were stained with hematoxylin–eosin (H&E) as previously described [64]. Histological examination was performed by a pathologist and scientists using a light microscope with an internal digital camera [65].

#### 4.6.2. Oil Red O Staining and Determination of Hepatic Lipid Content

Frozen liver sections (7-µm) were fixed with 5% paraformaldehyde and briefly incubated in 60% (*v*/*v*) isopropanol and exposed to Oil Red O (ORO) solution for 15 min in the dark. Sections were counterstained with hematoxylin, prior to imaging. For biochemical assessment of lipids, hepatic lipids were extracted using the method of Folch [66] with slight modifications, and triglycerides and cholesterol levels were quantified as described before [28].

#### 4.6.3. Periodic Acid Schiff (PAS)

Frozen liver sections (7-µm) were fixed with 5% formalin, washed, and oxidized with 1% (*w*/*v*) Periodic acid for 15 min. After washing, the slides were placed in Schiff’s reagent (Merck, Darmstadt, Germany) for 30 min as described [67], and were counterstained by Mayer’s Hemalum solution (Merck). For quantitative analysis, the percentage of PAS^+^ cells per high-power field (×200) to the total number of hepatocytes was determined using five random microscopic fields per animal.

### 4.7. Hepatic and Pancreatic RNA Isolation Real-Time Reverse Transcription-PCR

Total RNA was extracted from rat liver by Trizol reagent (Life Technologies, Carlsbad, CA, USA). RNA concentration and integrity were determined as previously described [65]. The primer sequences (Invitrogen, Karlsruhe, Germany) that utilized in this study are listed in Table S1.

Between 25 and 30 g pancreatic tissue was used for RNA extraction using density gradient purification [68]. Briefly, fresh samples were placed in cold guanidine-thiocyanate and β-2-Mercaptoethanol. After homogenization, samples were loaded gently on CsCl before centrifugation at 20 °C for 18 h at 35,000 rpm. The pellet was collected, air-dried, and re-suspended with de-ionized water. Samples were mixed with 5.6 mM Na-acetate and ethanol before storage for 1 h at −80 °C. After 30 min centrifugation at 4 °C, the pellet was dried using Vacuum Concentrator for 10 min, re-suspended in 100 µL de-ionized water, and heated for two min at 56 °C. After measuring the RNA concentrations, cDNA of all samples was generated by reverse transcription from 1.0 µg of total RNA by using the StepOne Plus system [65]. Primers used to detect *Insulin-1* and *glucagon* are shown in Table S1. β-actin (*Actb*) and ubiquitin C (*Ubc*) were designed as endogenous references (housekeeping genes). The results were normalized to the LFD (chow) group, and relative fold change of the gene expression was calculated using the 2^−ΔΔ*C*t^ method.

### 4.8. Western Immunoblots for Hepatic Extracts

A 100 mg liver sample from each animal was homogenized in RIPA buffer and the total protein concentration was determined using the Bradford method (Pierce, Rockford, IL, USA). Fifty milligrams of proteins were separated by 4%–12% SDS-PAGE and transferred to nitrocellulose membrane as described before [26]. Antibodies for immunoblotting are listed in Table S2. Immunoreaction signals were viewed with enhanced chemiluminescence (GE Healthcare, Munich, Germany) using a film processor machine. ImageJ 1.46 s (NIH, Bethesda, MD, USA) was used for the densitometric analyses.

### 4.9. Statistical Analysis

Values are expressed as means ± SEM. Statistical analysis was performed with ANOVA followed by Tukey’s protected least-significant difference test or Bonferroni test, where appropriate (GraphPad Prism 5 software, San Diego, CA, USA). Total cumulative glucose levels for each treatment group are reported as “areas under the curve” (AUC).

## 5. Conclusions

Feeding rats at different times intervals was useful to define the changes in BW and the features of MetS and insulin-resistance. Long-term hypercaloric consumption accompanied by a sedentary lifestyle predisposes T2DM, regardless of the amount of fat in the diet, as seen in the LFD and HFD. Not only is the nutritional value an important factor in predisposing diabetes and MetS, but the total energy intake is also significant. Merging alcohol-plus-fructose with HFD induced hepatomegaly, which is not related to changes in the BW and cannot be only explained as a result of the increased deposition of fat in hepatocytes. Intake of HF-EFr diet aggravated MetS, and impaired insulin clearance, emphasizing the pathogenic impacts of effects of concurrent alcohol and fructose consumption, and underscoring the urgent need for increased public awareness about the risks associated with consumption this diet. Life-style modifications, including physical activity and consumption of a Mediterranean diet, could be the first line of the treatment. ROS-induced ERK1/2 signaling and canonical NF-κB pathways in inflammation could be associated with impairment of hepatic insulin signaling. Whether other factors that increase liver weight induced by ethanol/ fructose consumption also reduce insulin-sensitivity of hepatocytes remains to be determined.

## Figures and Tables

**Table 1:** Diet preparation. The diet bases on purified feed ingredients and is intended for liquid feeding together with maltodextrin and/or ethanol/fructose.

Components	HFD		HF-EFr	
	Weight (g)	Energy (%)	Weight (g)	Energy (%)
LDC	132	637	83	400
Maltose dextrin	90	360	0	
Ethanol	0		42	299
Fructose	0		77	308
H_2_O	790		798	
Total	1012	997	1000	1007

Lieber-DeCarli crude (ssniff Spezialdiäten, Soest, Germany) contains micronutrients like amino acids, fat- and water-soluble vitamins, minerals, trace elements, un-/saturated FFAs, and fiber.

**Table 2:** Effect of each diet on plasma parameters in fasted state over eight weeks.

Parameters	Time Point	LFD	HFD	HF-EFr
Protein (g/dL)	Week 1	5.3 ± 0.1 ^¶,^*	5.5 ± 0.1	5.4 ± 0.1 *
	Week 4	5.9 ± 0.1	5.8 ± 0.1	5.9 ± 0.1
	Week 8	5.7 ± 0.0	5.9 ± 0.2	5.7 ± 0.1
Albumin (g/dL)	Week 1	3.91 ± 0.01	3.92 ± 0.01	3.80 ± 0.02 *^,*a,b*^
	Week 4	3.93 ± 0.01	3.83 ± 0.02	4.08 ± 0.08 *
	Week 8	3.90 ± 0.07	3.84 ± 0.06	3.72 ± 0.03 *^a,b^*
ALT (U/L)	Week 1	44 ± 3	36 ± 1	32 ± 2 ^¶,*a*^
	Week 4	50 ± 2	39 ± 1 *^a^*	69 ± 3 *^a,b^*
	Week 8	43 ± 2	36 ± 3	57 ± 2 *^a,b^*
AST (U/L)	Week 1	58 ± 1 *	61 ± 2	56 ± 1 ^¶,^*
	Week 4	73 ± 4	70 ± 2	87 ± 1 *^a,b^*
	Week 8	76 ± 2	63 ± 1	85 ± 2 *^b^*
Creatinine (mg/dL)	Week 1	0.18 ± 0.02 ^¶,^*	0.17 ± 0.00 ^¶,^*	0.15 ± 0.01 ^¶,^*
	Week 4	0.27 ± 0.01 *	0.25 ± 0.00 *	0.24 ± 0.02
	Week 8	0.31 ± 0.02	0.29 ± 0.01	0.27 ± 0.02
HOMA-IR	Week 1	0.61 ± 0.02 *	0.72 ± 0.10 ^¶^	0.88 ± 0.03 ^¶,^*^,*a*^
	Week 4	0.71 ± 0.08 *	2.85 ± 0.05 *^,*a*^	6.31 ± 0.75 *^,*a,b*^
	Week 8	2.01 ± 0.13	0.78 ± 0.12 *^a^*	3.70 ± 0.35 *^a,b^*
QUICKI	Week 1	0.42 ± 0.01 *	0.41 ± 0.01 ^¶^	0.39 ± 0.00 ^¶,^*^,*a*^
	Week 4	0.41 ± 0.01 *	0.33 ± 0.00 *^a^	0.29 ± 0.01 *^,*a,b*^
	Week 8	0.34 ± 0.01	0.41 ± 0.01 *^a^*	0.32 ± 0.00 *^a,b^*
Pancreatic amylase	Week 1	1748 ± 10 *	1955 ± 89	2003 ± 59 *
	Week 4	1630 ± 49	1971 ± 80 *^a^*	2125 ± 40 *^,*a*^
	Week 8	1482 ± 60	1695 ± 79	1774 ± 27 *^a^*
Leptin (ng/mL)	Week 1	2.0 ± 0.0 *	4.0 ± 0.7 *^a^*	4.0 ± 0.7 *^,*a*^
	Week 4	2.0 ± 0.2 *	3.0 ± 0.1 *^a^*	3.1 ± 0.3 *^,*a*^
	Week 8	3.6 ± 0.6	3.8 ± 0.6	9.1 ± 1.4 *^a,b^*

*^a^* indicates *p* < 0.05 vs. the LFD and *^b^* *p* < 0.05 vs. HFD group within the row, while ^¶^ significant difference vs. corresponding Week 4 of each group and * represents significant difference vs. corresponding Week 8 of the same group (columns). ALT: alanine transaminase; AST, aspartate transaminase; HOMA-IR, homeostatic model assessment–insulin resistance; QUICKI, quantitative insulin sensitivity check index. Data represent mean values ± standard error of six rats.

## Supplementary Materials

Supplementary materials can be found at www.mdpi.com/1422-0067/18/2/285/s1.
