# Supplementary material for: Insulin Production and Resistance in Different Models of Diet-Induced Obesity and Metabolic Syndrome
Source: Int J Mol Sci. 2017 Jan 28;18(2):285. doi: 10.3390/ijms18020285 (PMC5343821; doi:10.3390/ijms18020285)
Supplement: Supplementary file 1 [file ijms-18-00285-s001.pdf]

Supplementary Materials: Insulin Production and Resistance in Different Models of Diet-Induced Obesity and Metabolic Syndrome

Salamah Mohammad Alwahsh, Benjamin J. Dwyer, Shareen Forbes, David H. van Thiel, Philip Starkey Lewis and Giuliano Ramadori

Table S1. List of primers used in this study.

Gene	Sense 5′–3′	Antisense 5′–3′
Insulin-1	GGGGAACGTGGTTTCTTCTAC	GAGTGGTGGACTCAGTTGCAG
Glucagon	GAACGCCAGATCATTCCCA	GCAATGTTGTTCCGGTTCCT
Pik3ca	GTCACCATCAAGCATGAACTGT	GGTTGCCTACTGGCTCAATTAC
Fasn	TCGAGACACATCGTTTGAGC	CCCAGAGGGTGGTTGTTAGA
Cyp2e1	CCTACATGGATGCTGTGGTG	CTGGAAACTCATGGCTGTCA
Pparα	GTCATCACAGACACCCTCTC	CAGCTTCGATCACACTTGTC
Aco1	ATGGCAGTCCGGAGAATACCC	CCTCATAACGCTGGCTTCGAGT
β-actin	GAAATCGTGCGTGACATTAAAGAG	GCGGCAGTGGCCATCTC
Ubc	CACCAAGAAGGTCAAACAGGAA	AAGACACCTCCCCATCAAACC

Pik3ca: phosphoinositide-3-kinase, catalytic, α polypeptide, *Fasn*: fatty acid synthase, *Ubc*: ubiquitin c.

Table S2. List of antibodies used in this study.

Antibodies	Species	Reference	Dilution for WB
pERK1/2	Rabbit	Cell Signaling	1:800
tERK1/2	Rabbit	Cell Signaling	1:1000
IκB1α	Rabbit	Abcam	1:10,000
β-actin	Mouse	Sigma Aldrich	1:5000

WB: Western blot.

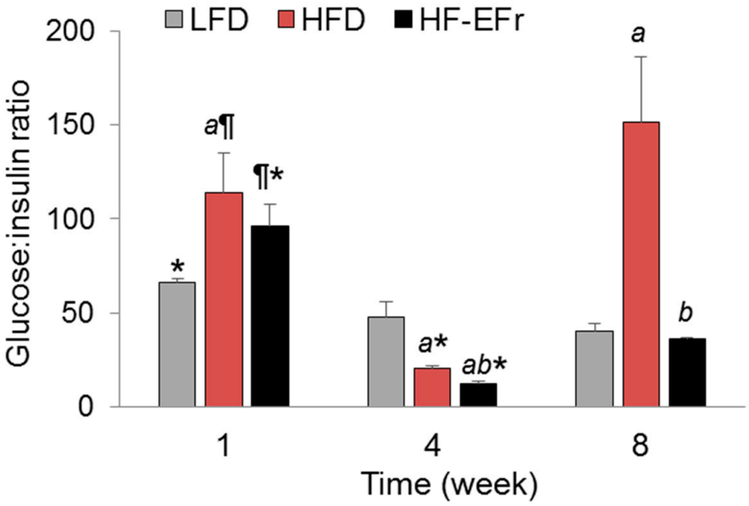

Figure S1. Fasting glucose-to-insulin ratio. [a] indicates a significant difference versus the LFD, [b] significant difference versus the HFD, ¶ indicates a significant difference versus Week 4 of within each group, and * significant difference versus Week 8 of within each group. LFD: low-fat-diet, HFD: high-fat-diet, HF-EFr: HFD + EtOH + Fructose.

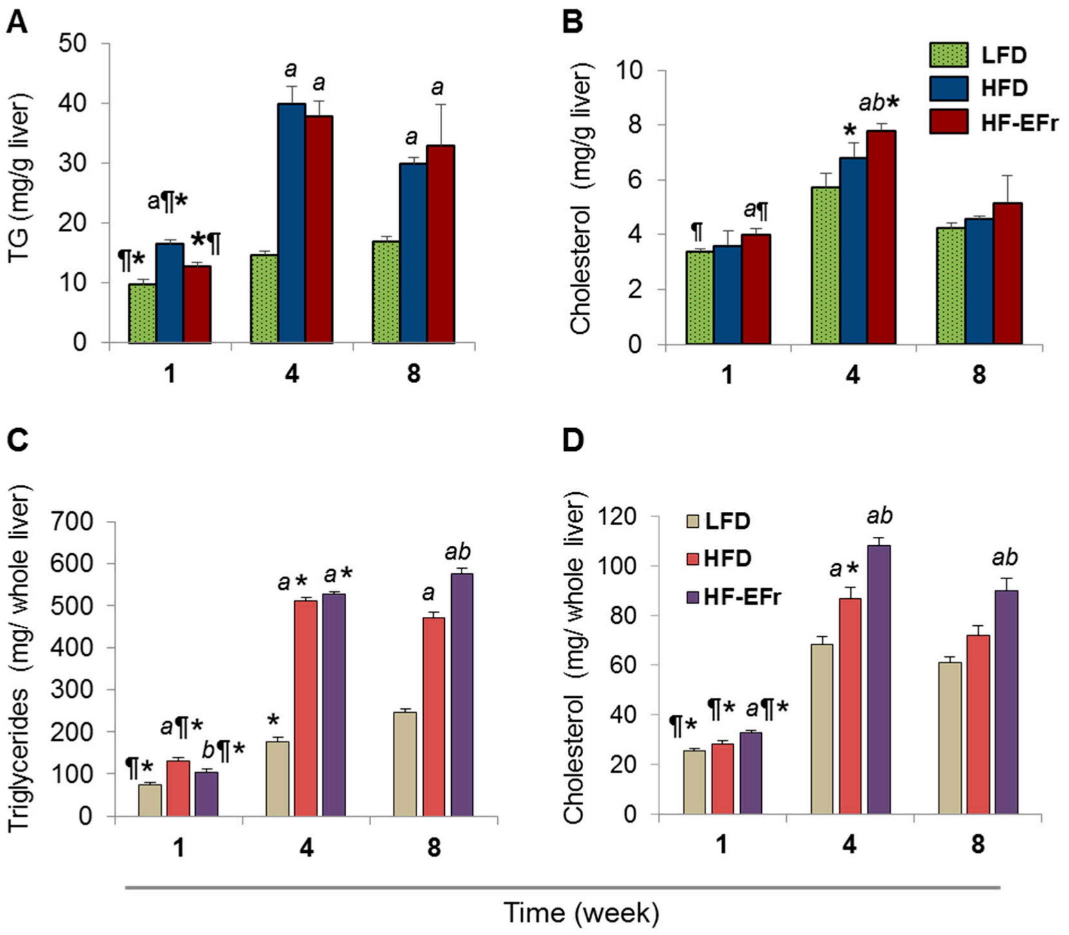

Figure S2. Changes in the amount of hepatic triglycerides and cholesterol among the groups at each time point. Concentrations of (**A**) intrahepatic triglyceride and (**B**) cholesterol per gram liver. Amount of total triglycerides(**C**) and cholesterol (**D**) per liver. [a] indicates a significant difference versus the LFD, [b] significant difference versus the HFD, ¶ indicates a significant difference versus Week 4 of within each group, and * significant difference versus Week 8 of within each group. We then calculated the total liver triglycerides and cholesterol content according to this formula: total triglycerides or cholesterol = amount of TG in mg/g liver × liver weight (g).

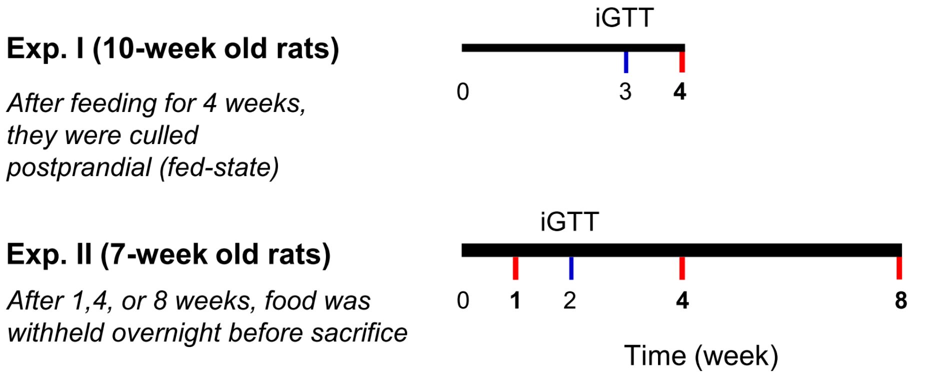

Figure S3. Timeline of the experimental design shows short-term feeding (one week) and long-term feeding until Week 8.
